# Methane Emission From Global Lakes: New Spatiotemporal Data and Observation‐Driven Modeling of Methane Dynamics Indicates Lower Emissions

**DOI:** 10.1029/2022JG006793

**Published:** 2022-07-27

**Authors:** Matthew S. Johnson, Elaine Matthews, Jinyang Du, Vanessa Genovese, David Bastviken

**Affiliations:** ^1^ Earth Science Division NASA Ames Research Center Moffett Field CA USA; ^2^ Bay Area Environmental Research Institute NASA Ames Research Center Moffett Field CA USA; ^3^ Numerical Terradynamic Simulation Group University of Montana Missoula MT USA; ^4^ California State University ‐ Monterey Bay NASA Ames Research Center Moffett Field CA USA; ^5^ Department of Thematic Studies ‐ Environmental Change Linköping University Linköping Sweden

**Keywords:** methane, carbon cycling, limnology, spatiotemporal, modeling, data sets

## Abstract

Lakes have been highlighted as one of the largest natural sources of the greenhouse gas methane (CH_4_) to the atmosphere. However, global estimates of lake CH_4_ fluxes over the last 20 years exhibit widely different results ranging from 6 to 185 Tg CH_4_ yr^−1^, which is to a large extent driven by differences in lake areas and thaw season lengths used. This has generated uncertainty regarding both lake fluxes and the global CH_4_ budget. This study constrains global lake water CH_4_ emissions by using new information on lake area and distribution and CH_4_ fluxes distinguished by major emission pathways; ecoclimatic lake type; satellite‐derived ice‐free emission period length; and diel‐ and temperature‐related seasonal flux corrections. We produced gridded data sets at 0.25° latitude × 0.25° longitude spatial resolution, representing daily emission estimates over a full annual climatological cycle, appropriate for use in global CH_4_ budget estimates, climate and Earth System Models, bottom‐up biogeochemical models, and top‐down inverse model simulations. Global lake CH_4_ fluxes are 41.6 ± 18.3 Tg CH_4_ yr^−1^ with approximately 50% of the flux contributed by tropical/subtropical lakes. Strong temperature‐dependent flux seasonality and satellite‐derived freeze/thaw dynamics limit emissions at high latitudes. The primary emission pathway for global annual lake fluxes is ebullition (23.4 Tg) followed by diffusion (14.1 Tg), ice‐out and spring water‐column turnover (3.1 Tg), and fall water‐column turnover (1.0 Tg). These results represent a major contribution to reconciling differences between bottom‐up and top‐town estimates of inland aquatic system emissions in the global CH_4_ budget.

## Key Points

- This study reports global, spatially‐ and temporally‐explicit lake CH_4_ emissions based on new spatiotemporal data
- Annual emission is 41.6 ± 18.3 Tg CH_4_: 14.1 Tg via diffusion, 23.4 Tg via ebullition, and 4.1 Tg from ice out and water‐column turnover
- This study addresses key issues in global lake emission estimates and reduces uncertainties in the global CH_4_ budget

## 1 Introduction

Lakes, defined as stable and non‐flowing water bodies which are open to the atmosphere (USGS, 2018), are a potentially important source of the greenhouse gas methane (CH_4_) to the atmosphere. However, estimates of lake emissions over large areas are very uncertain, and the spatiotemporal variability has not yet been fully considered in global estimates (Saunois et al., 2020). The first global estimate of CH_4_ emission from lakes, published almost 50 years ago (Ehhalt, 1974) based on two lake measurements and a global lake area of 2.5 × 10^6^ km^2^, reported that lakes may emit 1 to 25 Tg CH_4_ yr^−1^, the range reflecting different assumptions about the fraction of lakes emitting CH_4_ (Table 1). A later study (Bastviken et al., 2004) based on flux measurements from 74 lake systems reported that open water portions of lakes emit 6–25 Tg CH_4_ yr^−1^ (Table 1). Subsequent estimates, relying on data from ∼400 or more lake systems, reported 72 Tg CH_4_ yr^−1^ from diffusion and ebullition (Bastviken et al., 2011) and most recently 150 Tg CH_4_ yr^−1^ (mean) and 56 Tg CH_4_ yr^−1^ (median) (Rosentreter et al., 2021). This large range in estimates can be traced to differences in lake definition and areal extent, evolving availability and incorporation of different subsets of flux observations, definition of ice‐free/emission‐season length, and other methodological elements.

**Table 1:** Studies of Global CH_4_ Emission From Lakes

Study	System type	*N* lakes	Area (10^3^ km^2^)	Area source	Factors considered	Emission (Tg CH_4_ yr^−1^)					Flux pathway
						Min	Max	Mean	Median	Baseline estimate	
Ehalt et al. (1974)	Lake	2	2,500	1		1	25				
Smith and Lewis (1992)	Lake	17	2,500	2		11	55				
Bastviken et al. (2004)	Lake	73	2,800	3	Lake size; ice‐cover period estimated from air temp.	6	25				D, E
Bastviken et al. (2011)	Lake	397	3,740	4	Ice‐cover period estimated from air temperature; lake type/origin					72	D, E
Wik et al. (2016)^a^	Lake	733	1,840	5	Ice‐cover period estimated; lake type/origin, lake depth					16.5 ± 9.2	D, E, I
Holgerson and Raymond (2016)	Lake	427	5,822	6	CH_4_ flux and surface concentration					16	D
DelSontro et al. (2018)	Lake + Reservoir	561	3,230	7	Lake size/productivity relationships					104	D, E
DelSontro et al. (2018)	Lake + Reservoir	561	4,420	8	Lake size/productivity relationships					149	D, E
DelSontro et al. (2018)	Lake + Reservoir	561	5,129	9	Lake size/productivity relationships					185	D, E
Rosentreter et al. (2021)	Lake	227	3,856–6,551	10	Lake size; ice‐cover period estimated from air temp.			151	55.8		D, E, P
This study	Lake	575	2,800	See Methods Section	lake origin; ecoclimatic type; diel correction; modeled annual cycle of emissions; satellite‐derived freeze/thaw dynamics					41.6 ± 18.3	D, E, I, T

*Note.* Area source: 1. Hutchinson, 1948, 2. Wetzel, 2001, 3. Kalff, 2002, 4. Downing et al., 2006, excluding impoundments, rivers, and saline lakes; 5. Verpoorter et al., 2014. 6. Verpoorter et al., 2014 for lakes ≥0.001 km^2^ + modeled microlakes <0.001 km^2^; 7. Messager et al., 2016 + lakes 0.001–0.1 km^2^ + Caspian Sea; 8. Downing et al. (2006); 9. Verpoorter et al., 2014 + reservoirs (unknown source and area); 10. Loosely based on Verpoorter et al., 2014 + microlakes <0.001 km^2^. We note that unresolvable inconsistencies exist among lake areas reported by authors of the original lake data sets and those reported by authors of lake CH_4_ emission studies. Furthermore, undocumented alterations to lake data make comparisons of global lake areas used in individual studies challenging. Flux Pathways: Diffusion (*D*), Ebullition (*E*), Ice‐out (*I*), Water‐column turnover (*T*), Plant‐mediated transport (*P*).

^a^ Wik et al. (2016) only lakes >50°N.

More recent studies for estimating global lake CH_4_ emissions have attempted to add additional physiochemical drivers into their methods. Several studies have considered lake size (e.g., Bastviken et al., 2004; DelSontro et al., 2018) and/or defined emission seasons in very simple ways (Bastviken et al., 2004, 2011; Rosentreter et al., 2021; Wik et al., 2016). The recent study by DelSontro et al. (2018), combining lakes and reservoir emissions, used spatial patterns in chlorophyll‐a (Chl‐*a*, a proxy for primary productivity) as a driver of flux spatial variability (Table 1). Progress in further reducing uncertainties for improved global lake emission estimates has been stymied by several factors such as: (a) lack of systematic approaches to explicitly account for temporal and spatial flux variability among lake environments and seasons; (b) limited reliable data on lake area and distribution; (c) minimal observations of timing and duration of ice‐free/emission seasons; (d) not including ecoclimatic characteristics of lake systems; and (e) representativeness and utility of available flux observations. We also note that, to our knowledge, no current large‐scale, multi‐lake emission estimates are available in spatially‐ and temporally‐explicit formats.

Open water lake fluxes occur via multiple emission pathways regulated by a variety of drivers and processes. Most lake surface waters are supersaturated with CH_4_ relative to the atmosphere (Holgerson & Raymond, 2016; Rasilo et al., 2015), leading to fluxes across the water‐air interface. This emission of dissolved CH_4_ is the diffusive flux – named for the rate‐limiting transport across the water surface diffusive boundary layer. The concentration gradient across the water‐air interface is determined largely by surface‐water concentrations that are the net result of CH_4_ production, transport within the water‐column, consumption by CH_4_ oxidizing bacteria, and evasion rates (e.g., Bastviken et al., 2008). Ebullitive fluxes occur when CH_4_ bubbles formed in lake sediments are released and rapidly rise through the water‐column. Ebullition is regulated by organic matter input and CH_4_ production rates in the sediments, ease of bubble release, and pressure disturbances from currents, waves, or fluctuating barometric pressure (DelSontro et al., 2011, 2016; Eugster et al., 2011; Joyce & Jewell, 2003; Maeck et al., 2014; Mattson and Likens, 1993; Wik et al., 2018). Methane can also accumulate as bubbles in or under ice which can result in substantial emissions upon spring ice‐melt (Denfeld et al., 2018; Jansen et al., 2019) hereinafter referred to as the ice out flux. Methane can also accumulate in anoxic water layers that sometimes form in stratified water bodies. Some of this stored CH_4_ can be released upon water‐column turnover. The episodic fluxes initiated by ice‐melt and water‐column turnover have rarely been directly quantified but appear to be highly variable among lakes and depend on the extent to which CH_4_ is produced and oxidized in the lake before emission (e.g., Mayr et al., 2020; Phelps et al., 1998).

In addition to episodic temporal flux variability associated with diffusion, ebullition, ice out, and water‐column turnover, other factors contribute to temporal flux variability. Diel variability in lake CH_4_ emission rates was recently highlighted by Sieczko et al. (2020). In addition, exponential temperature relationships with both ebullition and diffusion have been revealed in multiple independent studies (Aben et al., 2017; Natchimuthu et al., 2016; Wik et al., 2014; Yvon‐Durocher et al., 2014) which is consistent with temperature sensitivities of CH_4_ production in sediments (Marotta et al., 2014). Such temperature relationships enable modeling of the seasonal variability found in the few studies where flux measurements were made over multiple seasons (e.g., Natchimuthu et al., 2016; Utsumi et al., 1998; Wik at al., 2016). The presence and duration of ice cover is one of the most important factors determining the seasonality of high‐latitude CH_4_ emission to the atmosphere. The length of the emission season has, to date, been treated in simple ways, that is, assuming a constant length for lake types (Wik et al., 2016) or defining emission seasons as those areas >0°C (Rosentreter et al., 2021). While lakes with continuous and very high ebullition can prevent ice formation locally and allow ebullition during winter (Walter Anthony & Anthony, 2013), ice formation seems to block most CH_4_ emissions during the winter.

Spatial variability in CH_4_ flux magnitudes among lakes has been associated with ecoclimatic lake types based on lake origin, soil type, permafrost status, and ice content of soils which are environmental variables likely influencing fluxes either directly or indirectly (Wik et al., 2016). Latitudinal zones have also been used to group lakes in past studies (e.g., Bastviken et al., 2011). While the relationships between lake CH_4_ fluxes and environmental variables are still not fully understood, classifying lakes by ecoclimatic region can account for some of the spatial flux variability under the assumption that existing observations implicitly represent regional lake characteristics.

A substantial range in estimates of CH_4_ emission from lakes is associated with different lake areas and distribution used among studies. Areas reported in lake studies over the last two decades range from 2.8 to 5.8 × 10^6^ km^2^ (Table 1). Some flux estimates were based on areas including both reservoirs and lakes (DelSontro et al., 2018); others incorporate modeled large areas of small lakes 0.001–0.1 km^2^ (Downing et al., 2006) or <0.001 km^2^ (Holgerson & Raymond, 2016). Several lake CH_4_ flux estimates depend on Verpoorter et al. (2014) lake areas derived from remote‐sensing approaches that can detect surface water but does not distinguish among aquatic features such as lakes, reservoirs, flooded wetlands, and rivers, and thus overestimates areas of lakes (Table 1) (DelSontro et al., 2018; Holgerson & Raymond, 2016; Rosentreter et al., 2021). Areal differences embedded in global lake emission estimates have been quantified using a single set of flux observations with three lake area data sets (DelSontro et al., 2018) (Table 1). The impact on CH_4_ emission of areal overestimates for high‐latitude lakes was identified by Matthews et al. (2020).

This study reports on a global estimate of temporally‐ and spatially‐explicit daily CH_4_ emissions from lakes using: (a) a new global data set of lake area and distribution; (b) diel corrections to flux observations; (c) seasonal flux variations modeled from temperature; (d) satellite‐derived ice‐free/emission‐season timing and duration; and (e) spatiotemporal flux variability associated with ecoclimatic lake types. We report daily climatological CH_4_ emissions over a full annual cycle representative of average conditions for 2003–2015. The goal of producing this spatially‐ and temporally‐explicit data is for future use in bottom‐up biogeochemical models, top‐down inverse model flux estimates, CH_4_ budget studies, and global climate and Earth System Models (ESMs). This study complements our recent research on global reservoir areas and daily CH_4_ emission gridded at the same 0.25° latitude × 0.25° longitude resolution (Johnson et al., 2021). The data sets are mutually‐exclusive and thus support assessment of the contribution of these separate, but related, sources to the global CH_4_ budget.

## 2 Methods

For consistency, all data sets associated with this new study are produced globally at 0.25° latitude × 0.25° longitude spatial resolution. For reference, a 0.25° × 0.25° grid cell is ∼750 km^2^ at the equator and ∼500 km^2^ at 50°N.

### 2.1 Flux Compilation, Augmentation, Correction

The flux compilation derived here is based on published data sets (Bastviken et al., 2011; Li et al., 2020; Rinta et al., 2017; Wik et al., 2016). We followed the approach to data processing described by Johnson et al. (2021) for reservoir CH_4_ flux measurements. Original references in each compilation were reviewed for every measurement to confirm or correct fluxes, and to extract information on measurement technique, water and/or air temperature contemporaneous with flux measurements, measurement time and duration (daytime only or 24‐hr measurements), and time of year (month of observation) (Johnson et al., 2021). Where multiple flux observations were reported as single averages, we expanded the compilation to reflect each observation when possible (i.e., when individual observations were reported). Subsequently, data were filtered to exclude indirect measurements (i.e., acoustic methods) and those lacking the necessary information on the time of day and month of year of observation and flux pathway (i.e., diffusive or ebullitive) (Johnson et al., 2021). For the boreal lake data provided in Wik et al. (2016) we removed systems identified as Beaver Ponds. After literature reassessment and filtering, data from 575 individual lake systems and 881 aggregated flux values (674 diffusion; 207 ebullition) were employed in our study (see Data Set S1; spatial distribution of measurement locations shown in Figure S1 in Supporting Information S1). Lastly, each flux measurement was classified into one of seven ecoclimatic lake types (see Section 2.3 below).

The lake CH_4_ flux compilation compiled for this study reflects the same limitations associated with the published data sets it is built upon. Specifically, ecoclimatic lake type emission rates are all derived from the limited data available. All high‐latitude lake types have monthly‐mean daily emission rates which are derived from <100 aggregated measurements (see Table 2). It should be noted that some of these aggregated measurements are averages of numerous individual measurements from multiple coincident lake/pond systems over extended time periods. As noted above, we disaggregated these measurements whenever possible. From Table 2 and Figure S1 in Supporting Information S1 it is clear that the majority of observation locations are in the Northern Hemisphere and primarily in temperate and high latitude regions. However, since the high latitudes are home to a more varied suite of lake types compared to other regions, individual high‐latitude lake types remain poorly represented in the observations. Moreover, our classification of tropical/subtropical lakes is currently very simple; a more nuanced classification would reveal how well these measurements represent tropical/subtropical lake types. It should be noted, given the lack of spatiotemporal coverage of daily lake CH_4_ flux observations available for application in global emission estimates, especially for ebullition fluxes which have large temporal variability, that this data set was not designed to study daily flux variability from individual lakes. This data set is designed for regional and global assessment of lake CH_4_ emissions and represents daily flux variability to the best of our ability given the observational data available. This is explained in the following sections.

**Table 2:** Area, Classification Criteria, Emission‐Season Length, and CH_4_ Emission Totals

Lake type	*N* ^a^	Area (×10^3^ km^2^)		SOC^b^ (kgC m^2^)	Permafrost category^c^	Ground‐ice (% volume)	Mean emission season (days)		Mean daily emission‐season flux (mg m^−2^)		Annual emission (Tg)
		<5,000 km^2^	≥5,000 km^2^				<5,000 km^2^	≥5,000 km^2^	<5,000 km^2^	≥5,000 km^2^	
High Latitude											
Thermokarst	70/17	234	0	≥0	C, D	≥10	107	N/A	80	N/A	2.0
Glacial/Postglacial	66/30	357	0	≥0	C, D	<10	117	N/A	31	N/A	1.3
Peat pond	42/1^d^	69	0	≥10	S, I	≥10	167	N/A	94	N/A	1.1
Organic	^e^	49	0	≥10	S, I	<10	183	N/A	89	N/A	0.8
Other Boreal	130/4	407	218	<10	None	None	152	135	65	7	4.2
Temperate	280/121	427	674	<10	None	None	289	206	65	9	9.3
Trop./Subtrop.	86/34	204	167	<10	None	None	363	365	235	23	18.8
		1,747	1,059								
Total	881	2,806									
Total *D* + *E*											37.5
Ice out + spring turnover											3.1
Fall turnover											1.0
Total emission											41.6

^a^ Number of aggregated flux measurements used to derive ecoclimate lake type monthly‐mean daily flux rates. Presented as (number of diffusion measurements/number of ebullition measurements).

^b^ Soil organic carbon, depth‐weighted to 1 m.

^c^ C, continuous; D, discontinuous; S, sporadic; I, isolated.

^d^ The single averaged value reported for the ebullition flux of peat ponds was based on measurements from seven different times between June–August in 15 pond systems, being widely distributed spatially (Pelletier et al., 2007). Overall, >300 individual measurements were used to derive this averaged flux rate. This illustrates that substantial measurement efforts can be included in producing a single aggregated measurement value, in our context being reported as *N* = 1.

^e^ Peat pond measurement data used to derive organic lake emission rates.

### 2.2 Satellite Observations of Timing and Duration of Ice‐Free Emission Season

We incorporated ice‐cover‐regulated emission seasonality using satellite microwave observations of ice‐cover phenology (Du et al., 2017; Du & Kimball, 2018) and freeze‐thaw dynamics (Kim, Kimball, Glassy, & Du, 2017; Kim, Kimball, Glassy, & McDonald, 2017; version 4 (FTv04)) as described in Johnson et al. (2021) and Matthews et al. (2020). These data sets of ice‐cover phenology and freeze‐thaw dynamics are provided at a daily frequency and ∼5 × 5 km^2^ and ∼25 × 25 km^2^ horizontal resolutions, respectively. To match the coarser spatial resolution of FTv04, and to remain consistent with spatial resolutions used in bottom‐up biogeochemical models, CH_4_ budget studies, and global climate and ESMs, we grid both satellite products at a 0.25° × 0.25° spatial resolution. Given that lake freeze‐thaw dynamics is a major driver of daily and seasonal variability in regional and global lake CH_4_ emissions, we produce our data set at the daily and 0.25° × 0.25° resolution consistent with the satellite data products. Lake ice phenology may vary with lake size and other physical characteristics (Thornton et al., 2015). The lake ice phenology data set used in this study (Du & Kimball, 2018) records daily ice‐cover conditions for Northern Hemisphere lakes with area ≥50 km^2^ using direct satellite observations and has 95% temporal accuracy relative to ground‐based observations (Du et al., 2017). The ice conditions were derived based on the high sensitivity of microwave remote‐sensing to the different dielectric properties of water and ice (Du et al., 2017). For relatively small lakes (surface area <50 km^2^) which are not represented in the ice‐phenology data set, daily landscape freeze/thaw dynamics of Kim et al. (Kim, Kimball, Glassy, & Du, 2017; Kim, Kimball, Glassy, & McDonald, 2017; FTv04) were applied for obtaining ice conditions. The FTv04 data set describes freeze/thaw conditions of the land surface consisting of different features such as bare land, vegetated land, rivers, and lakes. The data set shows classification accuracies of >84% relative to global weather station measurements and generally captures ice variation of small lakes as shown in the comparison with ice observations from the Global Lake and River Ice Phenology Database (Kim, Kimball, Glassy, & Du, 2017).

We calculated climatological conditions from both time series (lake‐ice phenology 2002–2015, freeze‐thaw 2003–2015). Local daily climatological thaw and freeze dates were derived by calculating mean thaw and freeze dates for each year and then averaged over the length of each data set. Climatological freeze/thaw dates were used to reflect typical conditions and maximize data available to define lake‐ice phenology and freeze‐thaw dynamics. The two satellite data sets were then combined to develop a complete year of global daily data that describes the timing and duration of ice‐free periods. For lakes which experience freeze/thaw, CH_4_ emissions commence on local thaw dates and end on local freeze dates (the difference between these dates defines emission‐season length). Mean emission‐season lengths for lake types are shown in Table 2.

### 2.3 Lake Area, Distribution, and Ecoclimatic Type

Several data sets of lake area and abundance have been published but only three are spatially explicit: Global Lakes and Wetlands Database (GLWD) (Lehner and Doll, 2004), HydroLAKES (Messager et al., 2016), and GLObal WAter BOdies database (GLOWABO) (Verpoorter et al., 2014). However, they are not in standard gridded form and thus are difficult to use as inputs to global bottom‐up biogeochemical and Earth System Models or top‐down inverse model approaches. Downing et al. (2006) provided statistics on lake area and abundance, by lake‐size classes and latitudinal bands, in tabular form combining GLWD data for lakes ≥10 km^2^ and modeled lakes down to 0.001 km^2^ using the Pareto distribution (Table 1); modeled small lakes and GLWD lakes >10 km^2^ each accounted for about 50% of total area. Thus, about half of the lake area in Downing et al. (2006) is the same as GLWD.

An important contributor to differences in lake areas (Table 1) is the minimum lake size represented in the data sets. The differences in these small lake areas are potentially important because small lakes are known to have high per m^2^ CH_4_ fluxes (Holgerson & Raymond, 2016; Rasilo et al., 2015). GLWD and HydroLAKES were developed using maps and modeling although random offsets in lake locations were identified in GLWD after publication. The smallest lake feature identified in both data sets is 0.1 km^2^. GLOWABO was produced from Landsat data including GeoCover (Thematic Mapper (TM)) and Landsat 7 Enhanced Thematic Mapper Plus (ETM+). These resources made it possible to identify lakes as small as 0.002 km^2^. However, the water class in GeoCover is described as “All types of water bodies, including rivers, lakes, reservoirs, ponds, bays, and estuaries. This categorization does not differentiate between these water classes.” In other words, remote‐sensing approaches generally capture all surface water (i.e., lakes, reservoirs, and rivers as well as flooded wetlands) without the ability to distinguish among them leading to overestimated areas.

**Figure 1:**
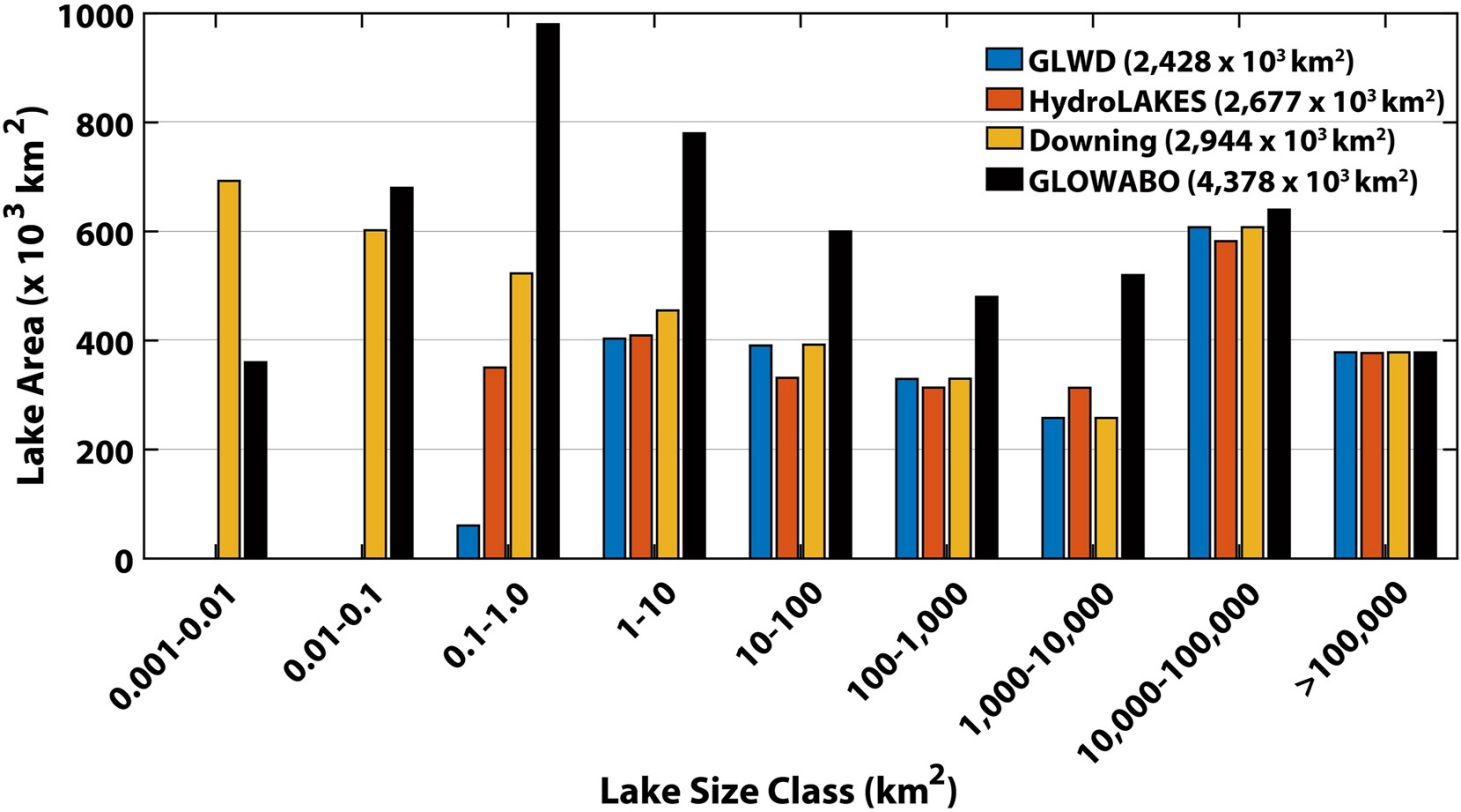
Global lake area (× 10^3^ km^2^), by size class, from the Global Lakes and Wetlands Database (GLWD), HydroLAKES, Downing et al. (2006), and GLObal WAter BOdies (GLOWABO) data sets. The figure legend presents the global total lake area, for all lakes >0.1 km^2^ (included in all data sets), for each data set.

We compared global lake areas, by size class, from the GLWD (Lehner & Doll, 2004), HydroLAKES (Messager et al., 2016), Downing et al. (2006), and GLOWABO/Verpoorter et al. (2014) data sets (see Figure 1). Areas are generally similar among all data sets for lake sizes >10,000 km^2^. However, for lakes between 0.1 and 10,000 km^2^, which all data sets report, GLOWABO is 1.5–2.8 times the areas from other data sets. Downing et al. (2006) and GLOWABO both report areas for the smallest lakes (0.001–0.1 km^2^) (1,300 and 1,040 × 10^3^ km^2^, respectively) and this is the only case in which GLOWABO areas are smaller than those in another data set. This could be due to the fact that Downing et al. (2006) models the areas of lakes in this size class. The difference between GLOWABO and the other data sets is most evident for lakes between 0.1 and 1.0 km^2^ where it is >16 times the area of GLWD. Furthermore, GLOWABO is between 1.9 and 2.8 times the areas from HydroLAKES and Downing et al. (2006) for lakes between 0.1 and 1.0 km^2^.

Comparing the global total area for lakes >0.1 km^2^ reveals that the GLOWABO area (4,378 × 10^3^ km^2^) is ∼50%–60% higher than Downing et al. (2006) (2,944 × 10^3^ km^2^) and HydroLAKES (2,677 × 10^3^ km^2^) and 80% larger than GLWD (2,428 × 10^3^ km^2^). If all lake sizes are included in the comparison, the GLOWABO global lake area total is 30% larger than Downing et al. (2006) and twice as large as HydroLAKES and GLWD. These differences suggest the strong, but rarely highlighted, potential influence exerted by the choice of lake area data on estimates of CH_4_ emission.

Our study required spatially‐explicit lake data. This, in addition to the undocumented random offsets in GLWD (determined by manual inspection/comparison with operational navigation charts) and identification of overestimates in the GLOWABO data set, determined that HydroLAKES was the only lake data set appropriate for this study.

**Figure 2:**
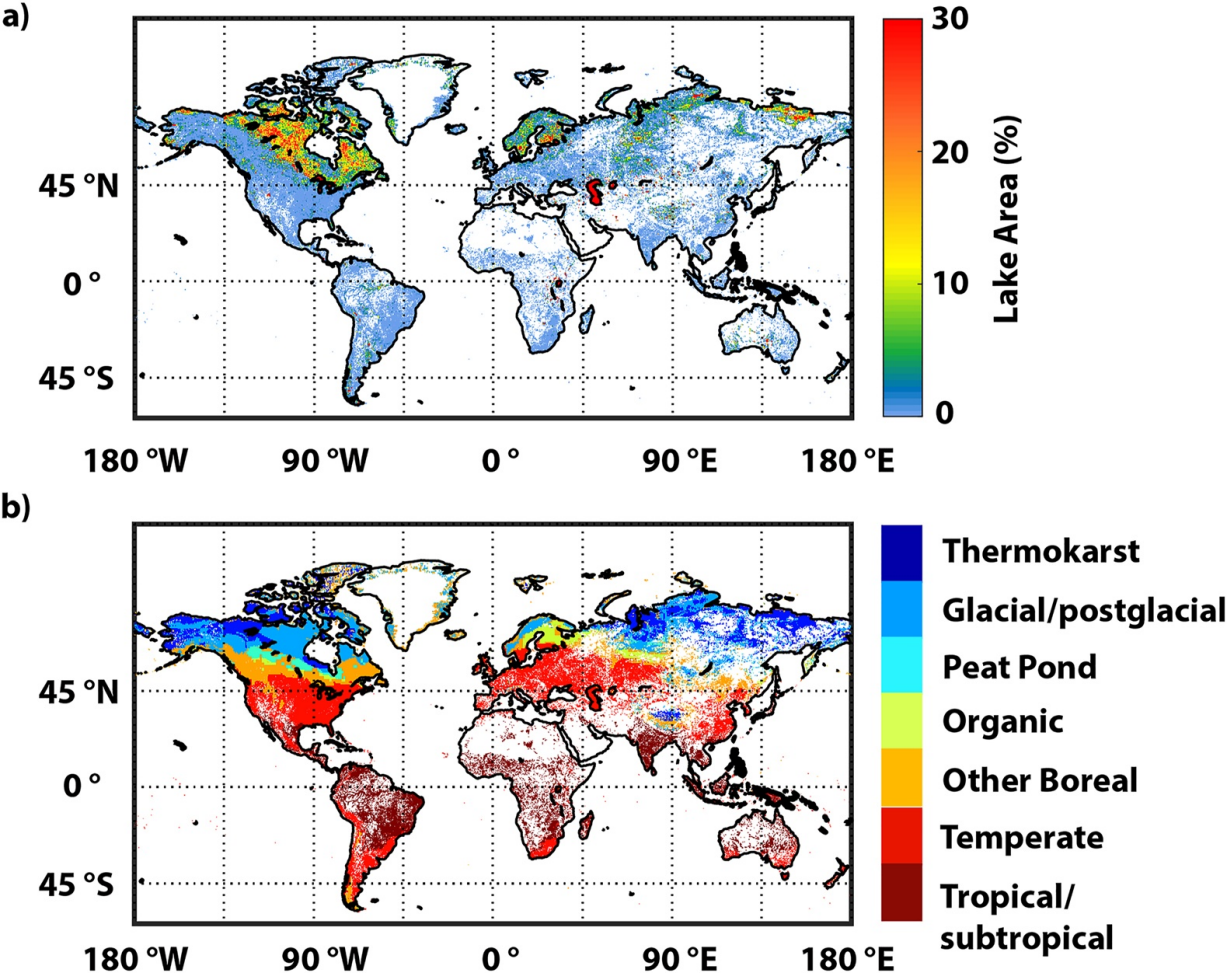
(a) Lake area density (% of grid cell area) and (b) ecoclimatic lake type classification. White space indicates grids with no lakes present.

Global lake area and spatial distribution for this study was extracted from HydroLAKES (Messager et al., 2016). The absence of lakes ≤0.1 km^2^ in HydroLAKES is an important limitation. We therefore augmented HydroLAKES with small lakes between 0.002 and 0.1 km^2^ extracted from the European Space Agency's Climate Change Initiative Inland‐Water (CCI‐IW) remote‐sensing data set (Lamarche et al., 2017) after removing non‐lake water bodies to isolate lakes only. River areas were removed from CCI‐IW using the Global River Widths (GRWL) data derived from Landsat (Allen & Pavelsky, 2018) and reservoirs were removed using our new reservoir data set (Johnson et al., 2021). Table 2 shows areas for lake types and Figure 2a shows the distribution of lake area density (lake fraction of grid cells).

Lakes were classified into ecoclimatic regions to facilitate linking these types with ecosystem specific CH_4_ measurements in the flux compilation. Ecoclimatic regions were defined using spatially‐explicit data on controlling conditions and variables (permafrost and ground‐ice state, soil carbon, and annually‐averaged soil temperature from the Modern‐Era Retrospective analysis for Research and Applications, Version 2 [MERRA‐2, Gelaro et al., 2017]); lake classification methods described in detail in Matthews et al. (2020); see Table 2. Annual soil temperature thresholds were then employed for the final classification of lake types following Johnson et al. (2021). No lake systems in the flux data compilation are classified as organic; however, while we distinguish between peat pond and organic lake spatial distributions, the organic lake diffusive and ebullition emission rates are derived from peat pond flux observations.

### 2.4 Lake Methane Fluxes

#### 2.4.1 Temperature‐Dependent Methane‐Flux Seasonality

The majority of measurements in our synthesis were made from late spring to early fall. Typically, past studies applied these fluxes throughout the year and defined emission‐season lengths from simple assumptions or models. However, lake CH_4_ emission rates are positively correlated with air and water temperature (e.g., Aben et al., 2017; Jansen et al., 2019; Natchimuthu et al., 2016; Wik et al., 2014; Yvon‐Durocher et al., 2014) meaning that using only high‐emission‐season fluxes likely overestimates annual emissions.

We corrected for this observational bias by calculating the monthly fluxes for each measurement using methods similar to Harrison et al. (2021) and Prairie et al. (2017) and described in detail in Johnson et al. (2021). Briefly, relationships between air temperature and ebullition and diffusive fluxes, together with the seasonal cycle of air temperatures at each measurement site, allowed us to calculate monthly‐mean daily fluxes for every sampled system (temperature‐flux relationships presented in Text S1 in Supporting Information S1). These monthly‐mean daily fluxes were applied throughout the emission‐season to arrive at total annual fluxes.

#### 2.4.2 Diel Variability

Sieczko et al. (2020) demonstrated that CH_4_ fluxes from lakes during daytime hours are larger by 50%–100% than those in early morning and night. In studies to date, daytime measurements have typically been used to represent 24‐hr daily averages, thus overestimating CH_4_ emissions. Most measurements in our synthesis were made during the daytime. To correct for diel fluctuations in CH_4_ fluxes, we employed information on the time of day extracted for each measurement in the compilation. Specifically, daytime‐only measurements (i.e., between 7:00 a.m. and 8:00 p.m. local time) were multiplied by the diel scaling factor of 0.7 (representing the best diel correction factor estimate given available data which appears to be consistent across numerous studies and different latitudinal regions; Sieczko et al., 2020). Measurements made over 24‐hr intervals were used as reported.

It is challenging to estimate CH_4_ emission from global lakes at a daily temporal scale due to insufficient spatiotemporal flux data coverage and the lack of observations of the associated environment variables. Instead, our data set accounts for the general patterns of daily emissions for different lake types based on available remote‐sensing and in situ observations across variant time scales. To take advantage of the daily lake ice phenology data used in this study, monthly‐averaged, diel‐ and temperature‐corrected monthly‐averaged daily emission rates were interpolated (cubic spline interpolation) to daily values throughout the annual cycle.

#### 2.4.3 Exploratory Estimates of Ice Out and Water‐Column Turnover Fluxes

We include exploratory estimates of fluxes associated with ice out (which includes spring water‐column turnover) and with fall water‐column turnover. Most estimates of such fluxes are indirect and based on differences between amounts of CH_4_ stored in the water‐column before and after ice‐out and/or water‐column turnover, which may indicate large fluxes (e.g., Bastviken et al., 2004; Denfeld et al., 2018), but direct flux measurements are limited. Given the scarcity of direct measurement information on lake fluxes associated with ice out and water‐column turnover, we estimate these fluxes by assuming:

1. that ebullition rates, adjusted to deep water temperatures of 5°C (Aben et al., 2017; Jansen et al., 2019) reflect the CH_4_ production rates and release from sediments below ice in winter or bottom waters during summer stratification (5°C represents the maximum water density temperature plus an assumed addition of 1°C from metabolic heat);
2. a lag time of 60 ± 15 days after lake thaw and freeze dates before lake water dissolved O_2_ is depleted and CH_4_ accumulation in the water‐column can start (Jansen et al., 2019; Vachon et al., 2019; lag times reported to have a range from 45 to 75 days);
3. CH_4_ oxidation removes on average 75% and 89% of the accumulated CH_4_ before emission during ice out + spring water‐column turnover and fall water‐column turnover, respectively (Boereboom et al., 2012; Fernandez et al., 2014; Kankaala et al., 2007; Mayr et al., 2020; Natchimuthu et al., 2016; Schubert et al., 2012; Striegl and Michmerhuizen, 1998; Utsumi et al., 1998; Vachon et al., 2019; Zimmerman et al., 2021; percentages are the means from cited studies);
4. CH_4_ accumulated in the water‐column during the freeze period is emitted evenly ±7 days of the thaw date to represent both ice‐out flux and spring water‐column turnover. For the fall water‐column turnover flux, accumulated CH_4_ was assumed to be emitted evenly over the 7 days prior to the freeze date.

#### 2.4.4 Emission From Large Lakes (≥5,000 km^2^)

Large lakes emit less CH_4_ per m^2^ than do smaller lakes (Bastviken et al., 2004; Holgerson & Raymond, 2016), and lakes ≥5,000 km^2^ are represented by very few flux measurements. Consequently, applying mean fluxes primarily from measurements of lakes <5,000 km^2^ to larger lakes would overestimate global fluxes. One CH_4_ study applied an average flux from all lakes to large lakes after removing 450,000 km^2^ of large saline lakes which still likely resulted in overestimates of large lake fluxes (Bastviken et al., 2011). Other regional studies excluded lakes >5,000 km^2^ (Matthews et al., 2020; Wik et al., 2016). However, observations of low fluxes from Lake Ontario and Lake Erie (Chau et al., 1977; Howard et al., 1971; Townsend‐Small et al., 2016), and of CH_4_ supersaturation in Lake Michigan (Joung et al., 2019) and the Aral Sea (Izhitskaya et al., 2019), show that emissions from these large lakes should not be excluded entirely. Based on a limited sample size, relationships between combined ebullition and diffusive CH_4_ flux versus lake size (Bastviken et al., 2004) indicate that emissions per m^2^ from lakes ≥5,000 km^2^ are in the range of 0%–25% of emissions from smaller lakes, although data on ebullition, being much higher in smaller lakes, were very scarce in this study. Based on this range, and likely underestimation of ebullition in the study generating the 25% value, we assumed that lakes ≥5,000 km^2^ have CH_4_ emission rates that are 10% of the fluxes observed from lakes <5,000 km^2^ in comparable ecoclimatic regions. This represents a reasonable exploratory estimate given presently available data but requires evaluation by future studies.

#### 2.4.5 Emission Uncertainty

Uncertainty (ε) in our global lake CH_4_ emission estimate is calculated using Equation 1 and propagating the individual uncorrelated uncertainties from the coefficient of variation in the time‐ and temperature‐corrected diffusive and ebullitive emission measurements (*ε*_*v*_), soil temperature threshold between temperate and tropical/subtropical lakes (*ε*_*t*_), CH_4_ accumulation lag time for ice‐out (*ε*_ai_) and fall water‐column turnover flux *(ε*_af_), the oxidation fraction considered for accumulated CH_4_ (*ε*_ox_), and large lake (≥5,000 km^2^) emission scaling factors *(ε*_sf_) through Equation 1.

(1) $$\varepsilon =\;\sqrt{\varepsilon_{v}^{2}+\varepsilon_{t}^{2}+\varepsilon_{\text{ai}}^{2}+\varepsilon_{\text{af}}^{2}+\varepsilon_{\text{ox}}^{2}+\varepsilon_{\text{sf}}^{2}\;}$$

The coefficient of variation in the time‐ and temperature‐corrected diffusive and ebullitive emission measurements was calculated directly from the variability in the flux measurement compilation data. The uncertainty in the soil temperature threshold separating temperate and tropical/subtropical lakes was assumed to vary by 2.5°C around the mean of 20.0°C. The lag time for CH_4_ accumulation for calculating ice‐out and fall turnover fluxes was assumed to vary by 15 days around the mean lag times of 60 days, respectively. To quantify the uncertainty due to the oxidation fraction used to calculate ice‐out and fall turnover fluxes we varied this value from 0.5 to 0.99. Finally, to determine the uncertainty in global lake emissions due to the larger lakes (≥5,000 km^2^), we assume large lake emissions are 0%–25% of the fluxes observed from lakes <5,000 km^2^ in comparable ecoclimatic regions instead of 10% used in baseline estimates. Using each uncertainty components in Equation 1 allows for the quantification of the overall uncertainty in our global emission estimate.

## 3 Results and Discussion

### 3.1 Lake Area

**Figure 3:**
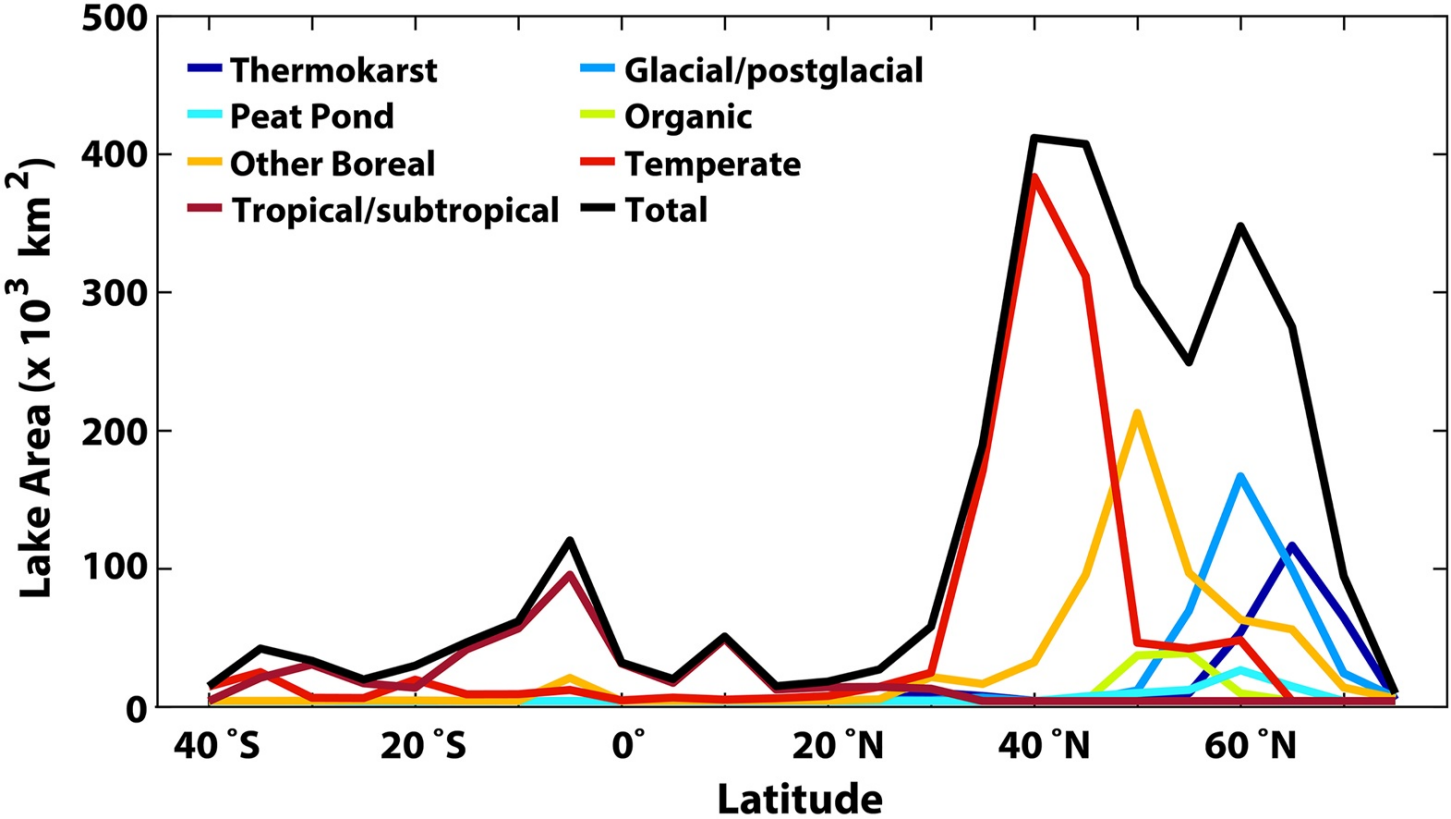
Zonal sums (5° latitudes, *x*‐axis label represents the southern limit of zones) of lake surface area (× 10^3^ km^2^) by ecoclimatic type.

Global lake area, from the merger of HydroLAKES (2,640 × 10^3^ km^2^) and smaller lakes from CCI‐IW (166 × 10^3^ km^2^), is estimated to be 2,806 × 10^3^ km^2^ (1,747 × 10^3^ and 1,059 × 10^3^ km^2^ for lakes < and ≥5,000 km^2^, respectively; see Table 2). The distribution of global lake area is shown in Figure 2a, and lake ecoclimatic types in Figure 2b; zonal lake areas, by type, are shown in Figure 3. Except for deserts and other arid environments, lakes occur throughout most of the world. Thermokarst, glacial/postglacial, peat pond, and organic lakes are prevalent in the high latitudes. About 50% of the global lake area is between 40° and 65°N where thermokarst, glacial/postglacial, other boreal, and temperate lakes exhibit large areal peaks. It is of importance for CH_4_ studies that the dense distribution of lakes in the high latitudes in North America and Siberia occurs within the same landscapes as CH_4_‐emitting natural wetlands, and that these different CH_4_ sources are poorly distinguished via remote‐sensing methods. Most wetland‐CH_4_ modeling studies (e.g., Melton et al., 2013; Wania et al., 2013) employ remotely‐sensed surface inundation to define wetlands meaning that lakes have been misallocated as wetlands while unflooded wetlands are not captured in the inundation data. Tropical/subtropical lakes exhibit a wide distribution of surface areas from 20°N to 30°S, with a modest peak between 10°S and the equator. Defining tropical lakes is particularly difficult since many lie along large rivers, and thus are distinct from rivers in dry seasons and engulfed by rivers during flood seasons.

### 3.2 Lake CH_4_ Emission

#### 3.2.1 Global CH_4_ Emission

Applying diel‐ and temperature‐corrected emission rates for ecoclimatic lake types (See Figure S2 in Supporting Information S1) over satellite‐derived ice‐free emission seasons, we estimate a global lake CH_4_ emission of 41.6 ± 18.3 Tg CH_4_ yr^−1^: 14.1 Tg CH_4_ yr^−1^ via diffusion, 23.4 Tg CH_4_ yr^−1^ via ebullition; ice out and spring water‐column turnover fluxes contribute another 3.1 Tg CH_4_ yr^−1^, and fall water‐column turnover adds 1.0 Tg CH_4_ yr^−1^. The total uncertainty is primarily from the large variability in the flux measurement data (66% of the total uncertainty) with smaller contributions from the definition of the temperate/tropical temperature threshold (11% of the total uncertainty), large lake emissions (14% of the total uncertainty), oxidation fractions applied in ice‐out and water‐column turnover emissions (7% of the total uncertainty), and ≤1% of the total uncertainty from ice out and fall water‐column turnover accumulation length assumptions. Tropical/subtropical diffusion and ebullition emission rates contributed the most to uncertainty due to variability in the flux measurement data. This is in part due to the limited amount of measurement data in this region where lake flux magnitudes are large and variable. Ice out and water‐column turnover emission estimates were consistent with the few representative direct measurements available (e.g., Erkkilä et al., 2018; Jammet et al., 2017; vs. corresponding latitudinal fluxes in Figure 5), and were regionally important but contributed <10% of the global total emission; therefore, we focus the discussion in the following sections primarily on the larger diffusion and ebullition (*D* + *E*) emissions.

**Figure 4:**
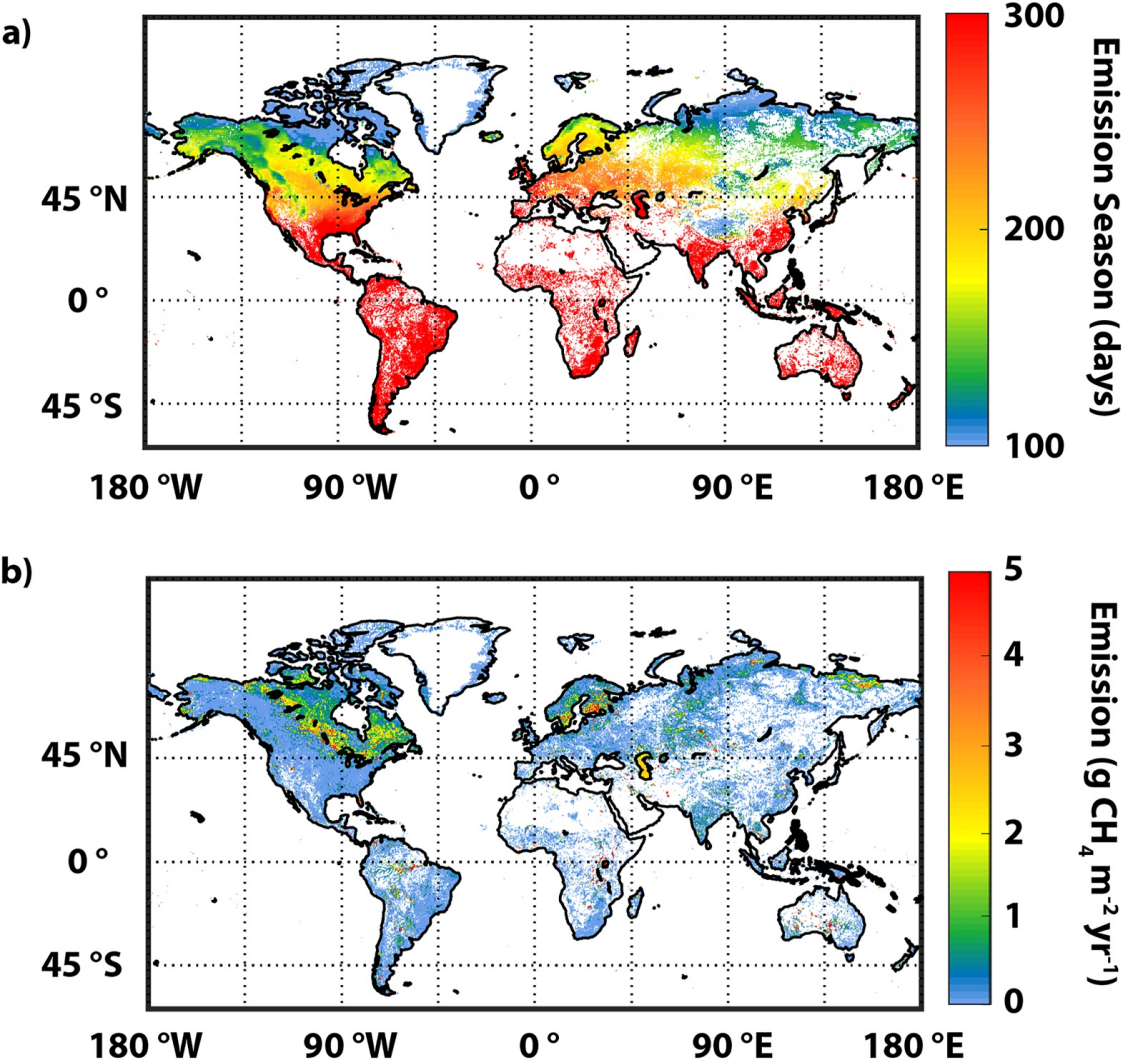
Global distribution of (a) emission‐season length (days) and (b) annual lake CH_4_ emission (gCH_4_ m^−2^ yr^−1^) via diffusive and ebullitive emission pathways. Note that Figure 4b shows lake emission as per m^2^ of grid cell. See Figure S4 in Supporting Information S1 which is similar to Figure 4b but showing lake emissions per m^2^ of lake in each grid cell.

The global distribution of annual *D* + *E* emissions is shown in Figure 4. For reference, the spatial distributions of annual ice out + spring turnover fluxes and fall turnover emission are shown in Figure S3 in Supporting Information S1. Our results for annual lake CH_4_ emission are higher than those reported before 2010 when flux data, particularly for ebullition, were scarce, but substantially lower than the global estimates published post‐2010 (Table 1).

**Figure 5:**
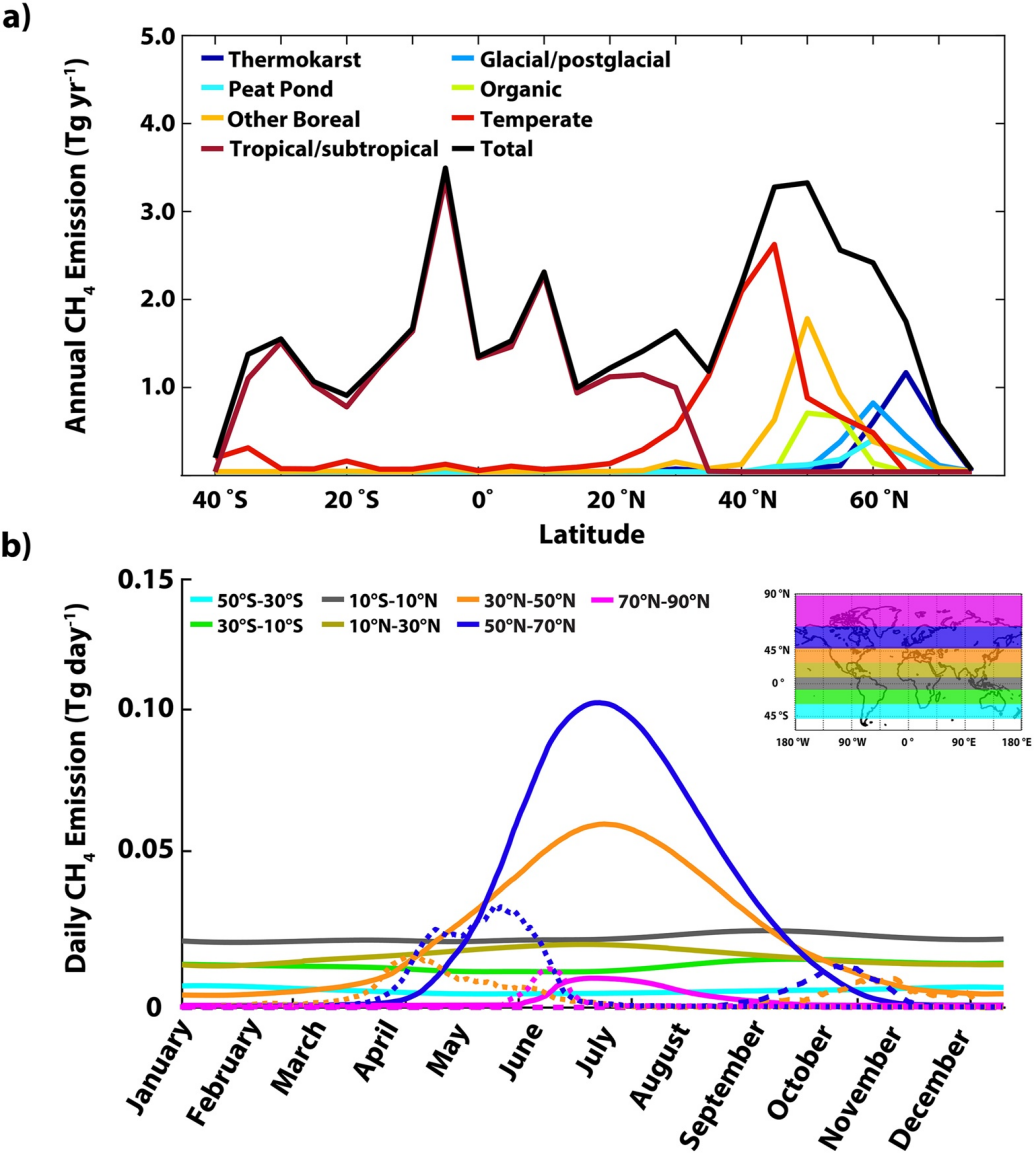
(a) Zonal sums (5° latitudes, *x*‐axis label represents the southern limit of zones) of annual lake emission (Tg yr^−1^) by ecoclimatic type and (b) 20° latitude zone sums (*x*‐axis label represents the southern limit of zones) of daily emissions (Tg day^−1^) from diffusion + ebullition (solid lines), ice out and spring water‐column turnover (dotted lines), and fall water‐column turnover flux (dashed lines). See Figure S2 in Supporting Information S1 for monthly‐mean daily emission rates (mg m^−2^ day^−1^).

Figure 5a shows zonal sums of annual CH_4_ emission by lake type. While 50% of lake area is between 40° and 65°N, these high‐latitude lakes contribute only ∼35% (15.1 Tg) to total annual emission due to abbreviated emission seasons (see Figure 3). Lakes in tropical/subtropical regions with long emission seasons and large *D* + *E* emission rates produce a broad band of substantial emissions between 20°S and 25°N while temperate lake emissions peak between 35° and 45°N.

#### 3.2.2 CH_4_ Emission by Ecoclimatic Lake Type

High‐latitude lake types (thermokarst, glacial/postglacial, peat pond, organic, and other boreal) are distributed throughout the northern regions and account for a modest 25% (9.4 Tg) of *D* + *E* annual emission. Global areas and emissions are low for both peat pond and organic lakes, whereas thermokarst and glacial/postglacial lakes, which together occupy 21% of total lake area, account for only 9% of *D* + *E* emissions due to short emission seasons (means of 110 ± 7 days) (see Table 2). Temperate lakes, concentrated in the United States, Europe, and China, contribute ∼25% (9.3 Tg CH_4_ yr^−1^) of global lake *D* + *E* emission. Lastly, 50% (18.8 Tg CH_4_ yr^−1^) of total *D* + *E* emission comes from tropical/subtropical lakes which are distributed throughout South America, Africa, India, Southeast Asia, and Australia.

This study is consistent with past studies reporting that ebullition is the dominant flux pathway (e.g., Wik et al., 2016) and that tropical areas contribute most to lake fluxes (e.g., Bastviken et al., 2011). Our ebullition emission rates for warmer climate lake types (i.e., tropical/subtropical and temperate lakes) are consistently higher compared to diffusion throughout all seasons (see Figure S2 in Supporting Information S1). Higher latitude lake types such as thermokarst, glacial/postglacial, and organic lake/peat ponds (except for the summer months for peat ponds) also display higher ebullition emission rates compared to diffusion. However, this is not consistent for all lake types, as other boreal lakes had higher diffusive emissions compared to ebullition in most months. Observations of ebullition indicate more frequent and larger fluxes at higher temperatures (in accordance with our temperature‐ebullition relationships; see Section 2.4.1 and Text S1 in Supporting Information S1). While ebullition fluxes are less frequent, and thereby more challenging to measure, in colder environments (Bastviken et al., 2011; Wik et al., 2016); it is vital that future flux measurements capture ebullition in representative ways in the colder temperate and boreal regions in order to avoid biased ebullition emissions in these regions. While fluxes associated with ice out and water‐column turnover can be large in some lakes, such fluxes are rather small in the global context.

#### 3.2.3 Methane Fluxes

Monthly mean daily emission‐season *D* + *E* CH_4_ fluxes for ecoclimatic lake types are shown in Table 2. Annual emission from large lakes ≥5,000 km^2^ is only 2.9 Tg (∼7% of the annual total). Therefore, the remainder of this section focuses on daily CH_4_ flux results from lakes <5,000 km^2^. Comparing mean daily emission‐season fluxes in Table 2 shows that all but tropical/subtropical and glacial lakes exhibit mean fluxes between 65 and 95 mg m^2^ day^−1^. Thus the wide range in total annual emission among lake types is controlled by surface area and length of the emission season. For example, mean daily fluxes and areas are similar for temperate and other boreal lakes, but temperate lakes account for 2.2 times the annual emission from boreal lakes because the mean temperate lake season length is twice that of the more northerly lakes.

#### 3.2.4 Length of Emission Seasons

Emission‐season length exerts a powerful influence on annual emissions in all systems outside of the tropics, although this controller is infrequently discussed. The spatial distribution of satellite‐derived ice‐free/emission‐season length is shown in Figure 3a and mean emission‐season fluxes are listed in Table 2. Mean CH_4_ emission seasons range from 107 days for thermokarst lakes to nearly 365 days for tropical/subtropical systems (Table 2).

Among high‐latitude systems, season length generally declines by ∼30 days with 10° increase in latitude (Figure 2) except for some continental and marine influences. Organic lakes, centered around 50°N, emit CH_4_ for an average of 183 days per year, whereas season lengths decline northward through other boreal lakes (152 days), glacial/postglacial lakes (117 days), and thermokarst lakes (107 days). A rough estimate of the impact of including realistic ice‐free emission seasons, based on information in Table 1, suggests that high‐latitude lakes would emit almost 2 to 3 times as much CH_4_ under the assumption of year‐round emissions.

#### 3.2.5 CH_4_ Emission Seasonality

Local, seasonally‐varying monthly‐averaged daily flux rates for lake types were applied to appropriate lake areas for the duration of the local thaw seasons. Mean emission‐season fluxes are listed in Table 2.

Largest emission rates occur between 50° and 70°N during the warm season that starts in early April and peaks at 0.1 Tg day^−1^ in June–July–August followed by a slow decline through late October (see Figure 5b). This seasonal cycle reflects the large lake areas and short emission seasons in the high latitudes. Temperate lakes emit the same amount of CH_4_ (9.3 vs. 9.4 Tg) annually as do high‐latitude lakes that occupy twice the area; however, temperate fluxes occur over a much longer thaw season averaging close to 290 days compared to high‐latitude lakes with season lengths of 107–183 days. Lower latitudes lakes show much less seasonality in emissions compared to the higher latitudes due to very low intra‐annual temperature fluctuations and freeze/thaw impacts.

## 4 Comparison With Other Lake CH_4_ Emission Estimates

Table 1 summarizes approaches and results for published estimates of global lake CH_4_ emission, including this study, and one study for lakes >50°N (Wik et al., 2016). Our estimate of 41.6 Tg CH_4_ yr^−1^ is lower than other recent studies that report global emissions of 56–185 Tg CH_4_ yr^−1^ (Table 1).

Global lake emission from our study (Table 1) is substantially lower than others due to the combined effects of a lower global area, diel‐ and seasonal temperature‐related flux corrections, and shorter emission seasons. The largest emissions for lakes, 150 Tg CH_4_ yr^−1^ (Rosentreter et al., 2021) and 185 Tg CH_4_ yr^−1^ (DelSontro et al., 2018), both relied on the GLOWABO lake data of Verpoorter et al. (2014) shown to overestimate global area compared to other data sets (Table 1; see Section 2.3). DelSontro et al. (2018) also includes an emission of unknown magnitude from reservoirs. Compared to the DelSontro et al. (2018) estimate using a similar area as we do, our global emission is lower due to the following: (a) our results are limited to lakes and DelSontro et al. (2018) includes reservoirs, (b) diel‐ and seasonality‐corrections are applied in our study which together reduce uncorrected emissions by almost 30%, (c) differences in upscaling approach – our study is anchored in flux observations and uses gridded data sets to define ecoclimatic regions while DelSontro et al. (2018) used Chl‐*a* to drive emissions, and (d) possible differences in length of emission periods; ours are driven by satellite data while DelSontro et al. (2018) does not report this variable.

Satellite‐observed ice‐free, emission‐seasons for lakes north of 50°N in our study are shorter by 11%–46% (18–54 days) than those assumed by Wik et al. (2016) for the same lake types. For lakes >50°N, Wik et al. (2016) reported a *D* + *E* + *I* emission of 16.5 ± 9.2 Tg CH_4_ yr^−1^ from 1840 × 10^3^ km^2^ of lakes, Matthews et al. (2020) reported 13.8–17.7 Tg CH_4_ yr^−1^ (*D* + *E* + *I*) from 1,095 × 10^3^ km^2^ of lakes, and our current study estimates 12.8 Tg CH_4_ yr^−1^ of *D* + *E* + *I* + *T* emissions from 1,260 × 10^3^ km^2^ of lakes. An inversion modeling study by Tan et al. (2016) for the pan‐Arctic region (>60°N) reported a priori lake emission of ∼11 Tg CH_4_ yr^−1^ derived from a processed‐based lake biogeochemical model (Tan et al., 2015), similar to that for Arctic thermokarst lakes only (Tan et al., 2015) indicating that all lakes >60°N are assumed to be thermokarst. However, Matthews et al. (2020) found that thermokarst lakes account for only 40% of pan‐Arctic lakes. This comparison suggests that CH_4_ estimates for high‐latitude lakes may be converging, but that emission‐season lengths, lake area, and lake‐type considerations are key for CH_4_ emission estimates and need careful consideration.

Accounting for diel emission cycles lowered our global *D* + *E* lake emission estimate by 8% or 4.2 Tg from a base *D* + *E* emission of 51.4 Tg with no corrections. Flux seasonality, driven by temperature‐corrected fluxes, further reduced fluxes by 21% (9.7 Tg). Together, diel‐ and temperature‐related seasonality corrections reduced total *D* + *E* uncorrected global emissions by nearly 30% and are a major contributor to our lower estimate compare to recent studies and confirms the importance of correcting for diel‐ and seasonal‐biases in flux measurements.

We devoted considerable effort to defining area and spatial distribution of lakes. To date, no studies explicitly incorporate the geographical distribution of lakes with the exception of the pan‐Arctic study of Tan et al. (2016); although areas and lake data used in Tan et al. (2016) are not reported. At best, past studies have relied on simple latitudinal assumptions. In contrast, our study merged two lake data sets, used multiple gridded geophysical variables (Table 2), and modeled air temperature from MERRA‐2 to define the spatial distribution of lakes and ecoclimatic types. This represents progress in defining CH_4_‐centric lake types that align with flux observations.

Our estimate is lower than recent emission estimates for identifiable and expected reasons as discussed above. Our findings also likely improve the quantification of the global CH_4_ budget. Recent work on the global CH_4_ budget highlights a large difference between unconstrained bottom‐up CH_4_ fluxes and top‐down inversion estimates of fluxes constrained by measurements of atmospheric CH_4_ concentrations (Saunois et al., 2020). Average global CH_4_ emission from bottom‐up and top‐down estimates for 2008–2017 are 737 and 576 Tg CH_4_ yr^−1^, respectively. Accounting for sinks and atmospheric growth, the imbalance is 112 Tg CH_4_ yr^−1^. However, Saunois et al. (2020) assumed that non‐wetland freshwater sources are 159 Tg CH_4_ yr^−1^. We constructed a new freshwater estimate of 79 Tg CH_4_ yr^−1^ consisting of 27 Tg CH_4_ yr^−1^ from rivers (Stanley et al., 2016) quoted in Saunois et al. (2020), 10 Tg CH_4_ yr^−1^ from reservoirs (Johnson et al., 2021), and our new lake estimate of 42 Tg CH_4_ yr^−1^. This revised freshwater aquatic emission estimate reduces the Saunois et al. (2020) bottom‐up total from 737 to 657 Tg CH_4_ yr^−1^, and the overall imbalance from 112 to 32 Tg CH_4_ yr^−1^. Therefore, this study contributes toward constraining both lake CH_4_ emissions and the global CH_4_ budget.

## 5 Prospects for Future Work

This study highlights several remaining problems that make reducing uncertainties in lake CH_4_ emissions challenging.

The abundance and area of small lakes <0.1 km^2^ with high per m^2^ emissions (e.g., Grinham et al., 2018; Holgerson & Raymond, 2016) remain poorly characterized. While high‐resolution remote‐sensing is capable of mapping small inland water bodies, it remains a major challenge to distinguish lakes from similar aquatic environments such as flooded wetlands, reservoirs, and other unidentified aquatic features.

Direct measurements of fluxes, including ebullition and emissions associated with ice out and water‐column turnover remain rare as they are costly and time consuming to make. Increasing the amount and frequency in which these direct observations are made would help reduce the uncertainty in extrapolating observations to global CH_4_ emission estimates. Moreover, studies over multiple seasons and covering spatial variability within and among lakes remain scarce, which is a major obstacle for model development and validation. Additional annual flux observations, taken throughout the diel cycle, from lakes of all types are needed. As noted previously, the limited spatiotemporal coverage of lake CH_4_ flux observations, in particular those for ebullition processes, hinders the ability for detailed upscaling global lake emissions data sets, such as the one produced in this study, to be applied for specific lakes on a daily‐basis. To study fine‐scale processes controlling individual lake's daily flux variability, applying a process‐level lake model, which incorporates hourly/daily input data of controlling variables, is a more appropriate tool (e.g., Tan et al., 2015, 2016). Finally, the limited information on diffusive and ebullition CH_4_ fluxes from very large lakes ≥5,000 km^2^ also represents an uncertainty that requires future attention.

There is also a particular shortage of detailed spatiotemporal studies of tropical lakes which account for about 50% of global lake emission. At low latitudes, small changes in temperatures can exert large absolute effects of CH_4_ emission (Gudasz et al., 2021; Marotta et al., 2014). Our study confirms anew the importance of low‐latitude lakes in the global CH_4_ budget. The observational base for these systems is sparse compared to high‐latitude lakes. Moreover, the dynamic and dominant association of low‐latitude lakes with large tropical river systems makes it difficult to unambiguously define lakes in these environments. Remote‐sensing studies such as that of Pekel et al. (2016) that identify seasonal and permanent water extents may contribute to resolving lake‐river dynamics in the tropics.

The length of ice‐free emission seasons quantified in this study is a primary determinant of total annual fluxes. Due to amplified warming in the high latitudes, a climate impact on future lake emissions north of the subtropics is expected, and is likely already occurring (Grant et al., 2021; Guo et al., 2020). Continued work to model northern lake fluxes, including lake initiation, evolution, drainage, and decline, and associated emissions, is critical to predicting future northern lake CH_4_ fluxes. Tan and Zhuang (2015) reported on modeling CH_4_ dynamics, as well as initiation and evolution of permafrost lakes. Expanding this approach to the full range of lake types would be a major step toward predicting future emission dynamics resulting from landscape evolution, especially in high‐latitude zones experiencing warming. Progress in understanding lake CH_4_ emissions will benefit from a diversity of modeling approachaes – some of which have already been initiated (e.g., Tan et al., 2015, 2016). However, these models will need substantially more systematic and representative data to reveal the regulation of different types of variability of the fluxes.

Lake productivity can exert strong influences on CH_4_ emission (e.g., Sepulveda‐Jauregui et al., 2018), implying that environmental change in catchments can have large impacts on emissions. Consequently, systematic monitoring of CH_4_ fluxes from lakes across all latitudes is important to constrain feedbacks of climate and environmental change. Overall, the prospect of long‐term changes in lake CH_4_ fluxes, and flux sensitivity to environmental change, calls for long‐term (decadal) monitoring of lake fluxes.

The compilation of flux observations developed for this study reveals that a large fraction of measurement data is not applicable for modeling lake emissions due to lack of ancillary information accompanying the measurements (e.g., time of day/year of measurement, measurement technique, identified flux pathway, etc.). Comprehensive site descriptions expand the use and value of flux observations.

## 6 Significance and Conclusions

We present a study based on a variety of observation‐based data sources and modeling approaches to develop a new global estimate of CH_4_ emission from lakes. Furthermore, this study generated a suite of global data sets at 0.25° resolution of lake area and distribution, ecoclimatic lake type, observed emission‐season timing and duration, diel and seasonal emissions patterns, and a full annual cycle of daily CH_4_ emissions. The spatial and temporal resolution of these data sets facilitates their use in bottom‐up biogeochemical models, top‐down atmospheric inverse models, climate models, and ESMs. The results are tightly anchored to field observations, in situ measurements, and remote‐sensing observations independent of diagnostic or prognostic models (e.g., ecosystem and/or biogeochemical models). This study highlights and quantifies the impact of corrections for diel and seasonal observational bias, observed ice‐free/emission seasonality, and realistic lake area and distribution that together explain the potential high biases in existing estimates of global CH_4_ emission from lakes. This research constrains global lake CH_4_ fluxes to 41.6 ± 18.3 Tg CH_4_ yr^−1^, reduces uncertainties in the global CH_4_ budget, and facilitates inclusion of lake CH_4_ fluxes in a wide range of biogeochemical and atmospheric models.

## Conflicts of Interest

The authors declare no conflicts of interest relevant to this study.

## Supporting information

Supporting Information S1

Data Set S1

## Data Availability

The gridded data sets produced in this study can be downloaded from NASA's Oak Ridge National Laboratory Distributed Active Archive Center (https://doi.org/10.3334/ORNLDAAC/2008). The lake CH_4_ flux compilation produced for this study is included with this manuscript as Data Set S1. The authors also acknowledge the usage of the publicly‐available MERRA‐2 meteorology and soil temperature data downloaded from NASA's EarthData repository (https://earthdata.nasa.gov/; last access: 11/10/2020). Data used for producing the remote‐sensing freeze/thaw information applied in this study were downloaded from NASA's National Snow and Ice Data Center Distributed Active Archive Center (https://doi.org/10.5067/HT4NQO7ZJF7M and https://doi.org/10.5067/MEASURES/CRYOSPHERE/nsidc-0477.004; last access: 12/01/2020).
