# Supplementary material for: Methane Emission From Global Lakes: New Spatiotemporal Data and Observation‐Driven Modeling of Methane Dynamics Indicates Lower Emissions
Source: J Geophys Res Biogeosci. 2022 Jul 27;127(7):e2022JG006793. doi: 10.1029/2022JG006793 (PMC9540782; doi:10.1029/2022JG006793)
Supplement: Supplementary file 1 — Supporting Information S1 [file JGRG-127-e2022JG006793-s002.docx]

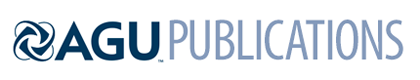


*Journal of Geophysical Research: Biogeosciences*

Supporting Information for

**Methane emission from global lakes: new spatiotemporal data and observation-driven modeling of methane dynamics indicates lower emissions**

Matthew S. Johnson^1^, Elaine Matthews^2^, Jinyang Du^3^, Vanessa Genovese^4^ and David Bastviken^5^

^1^NASA Ames Research Center, Moffett Field, CA, USA, matthew.s.johnson@nasa.gov.

^2^Bay Area Environmental Research Institute, NASA Ames Research Center, Moffett Field, CA, USA, matthews@baeri.org.

^3^Numerical Terradynamic Simulation Group, University of Montana, Missoula, Montana, USA, jinyang.du@mso.umt.edu.

^4^California State University - Monterey Bay, NASA Ames Research Center, Moffett Field, CA, USA, vanessa.genovese@nasa.gov.

^5^Department of Thematic Studies - Environmental Change, Linköping University, Linköping, Sweden, david.bastviken@liu.se.

**Contents of this file**

Supplemental Text S1.

Figures S1 through S4.

**Additional Supporting Information (Files uploaded separately)**

Data set S1

**Introduction**

All supplemental figures in this document are referenced in the main text of the manuscript. Figure S1 is included to show the spatial distribution of the locations of diffusive and ebullitive measurements incorporated in Data set S1. Figure S2 presents the monthly-averaged daily diffusive and ebullitive CH_4_ daily emission rates for thermokarst, glacial/postglacial, organic, peat pond, other boreal, temperate, and tropical/subtropical lakes derived in this study. These ecoclimate lake type emission rates were derived with Data set S1 which is attached as a separate document. Figure S3 shows the global spatial distribution of emissions from the flux pathways of ice out (includes spring water-column turnover fluxes) and fall water-column turnover emissions (per m^2^ of each grid cell). Finally, Fig. S4 shows the same image as Fig. 4 in the main manuscript (global distribution of D+E lake emissions) but with units of emissions per m^2^ of lake area in each grid cell.

**Supplemental Text S1**

**We refer the readers to Johnson et al. (2021), which is referenced in the main text of the manuscript, for the detailed description of how the temperature to ebullition and diffusion flux relationships were derived. Briefly, the temperature dependence of ebullitive fluxes is calculated applying a modified Arrhenius equation shown in Eq. (1)**

$\boldsymbol{E}_{\boldsymbol{B}}\boldsymbol{=}\boldsymbol{E}_{\boldsymbol{20}}\boldsymbol{\times}\boldsymbol{\theta}_{\boldsymbol{s}}^{\boldsymbol{(T-20)}}$ **(1)**

**where** $\boldsymbol{E}_{\boldsymbol{B}}$ **represents the ebullitive emission rate (mg CH_4_ m^-2^ day^-1^),** $\boldsymbol{E}_{\boldsymbol{20}}$ **is the emission rate at 20 °C,** $\boldsymbol{\theta}_{\boldsymbol{s}}$ **represents the system temperature coefficient, and *T* is surface air temperatures (°C). The temperature-diffusive flux regression fit is shown in Eq. (2)**

$\boldsymbol{E}_{\boldsymbol{D}}\boldsymbol{=0.023}\boldsymbol{e}^{\boldsymbol{0.124}\boldsymbol{T}}$ **(2)**

**where** $\boldsymbol{E}_{\boldsymbol{D}}$ **represents the diffusive emission rate (mg CH_4_ m^-2^ day^-1^).**


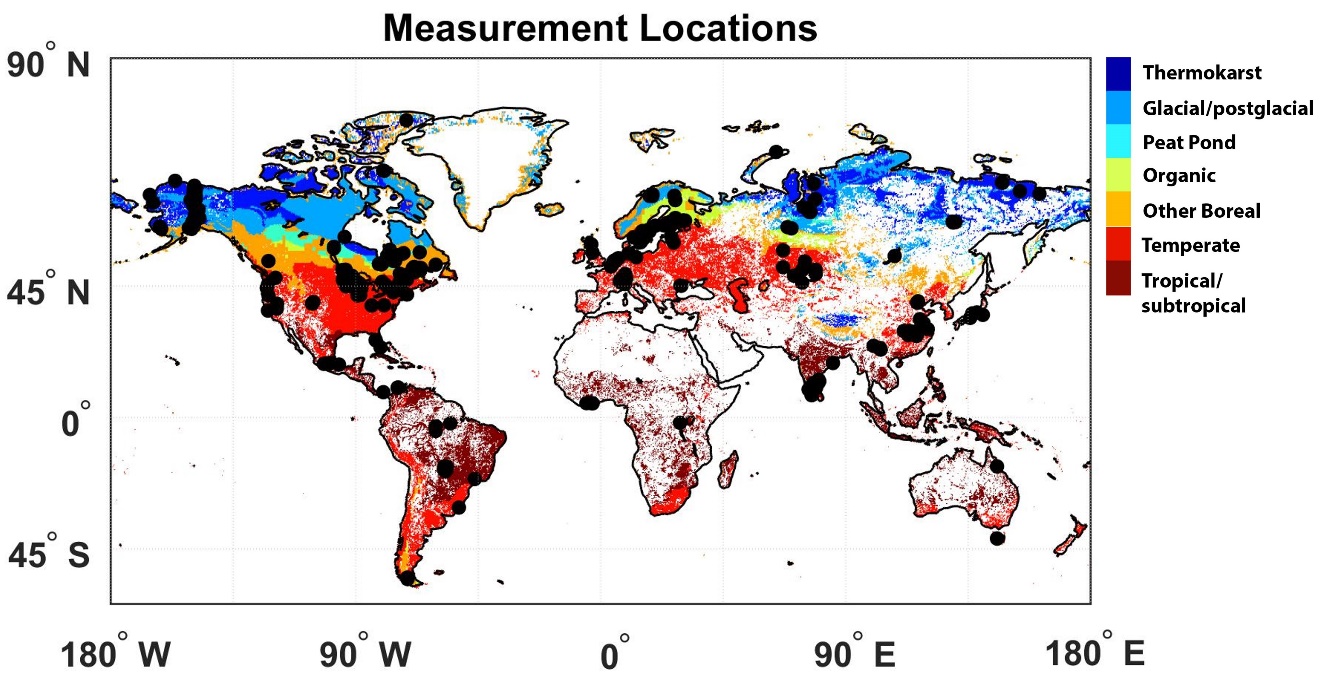


**Figure S1.** The locations of diffusive and ebullitive emission measurements (black circles) presented in Data Set S1. The measurement locations are overlayed on the map of ecoclimatic lake type in our data set.

**
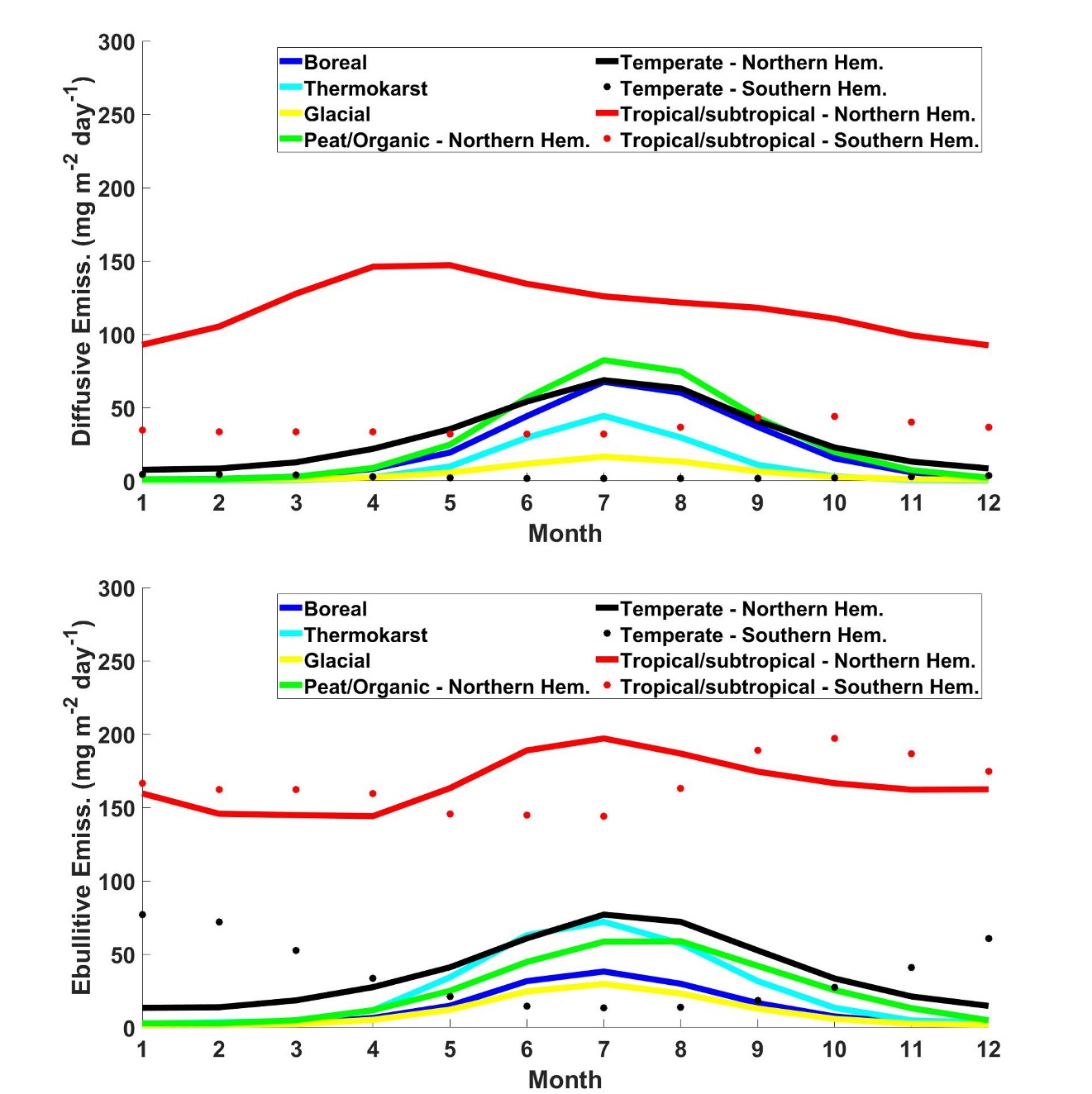
**

**Figure S2.** Monthly-mean daily diffusive (top) and ebullitive (bottom) emission rates (mg m^-2^ day^-1^) throughout the year.


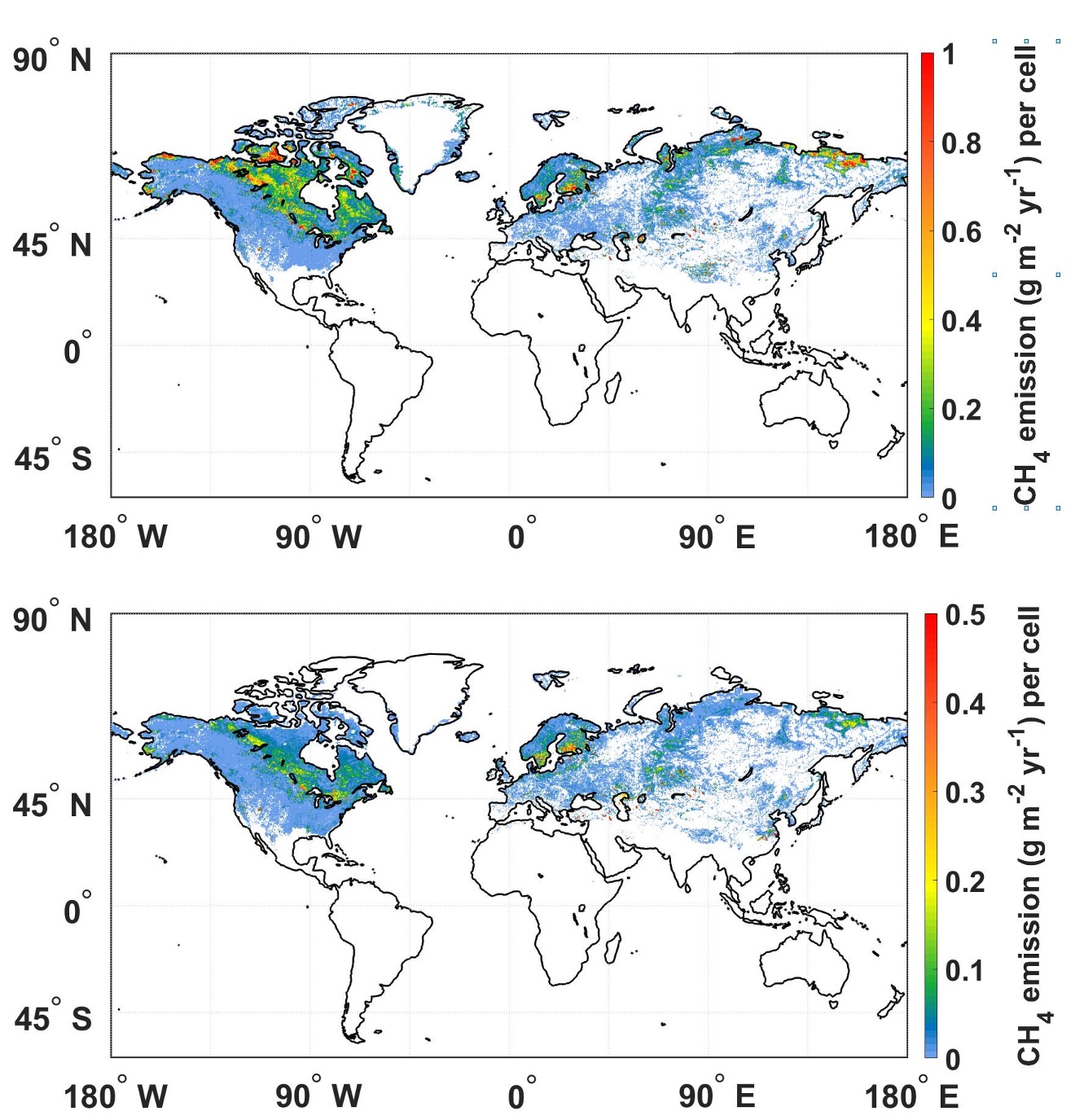


Figure S3. Global distribution of annual lake CH_4_ emission (gCH_4_ m^-2^ yr^-1^) from a) ice out (includes spring water-column turnover) and b) fall water-column turnover fluxes.


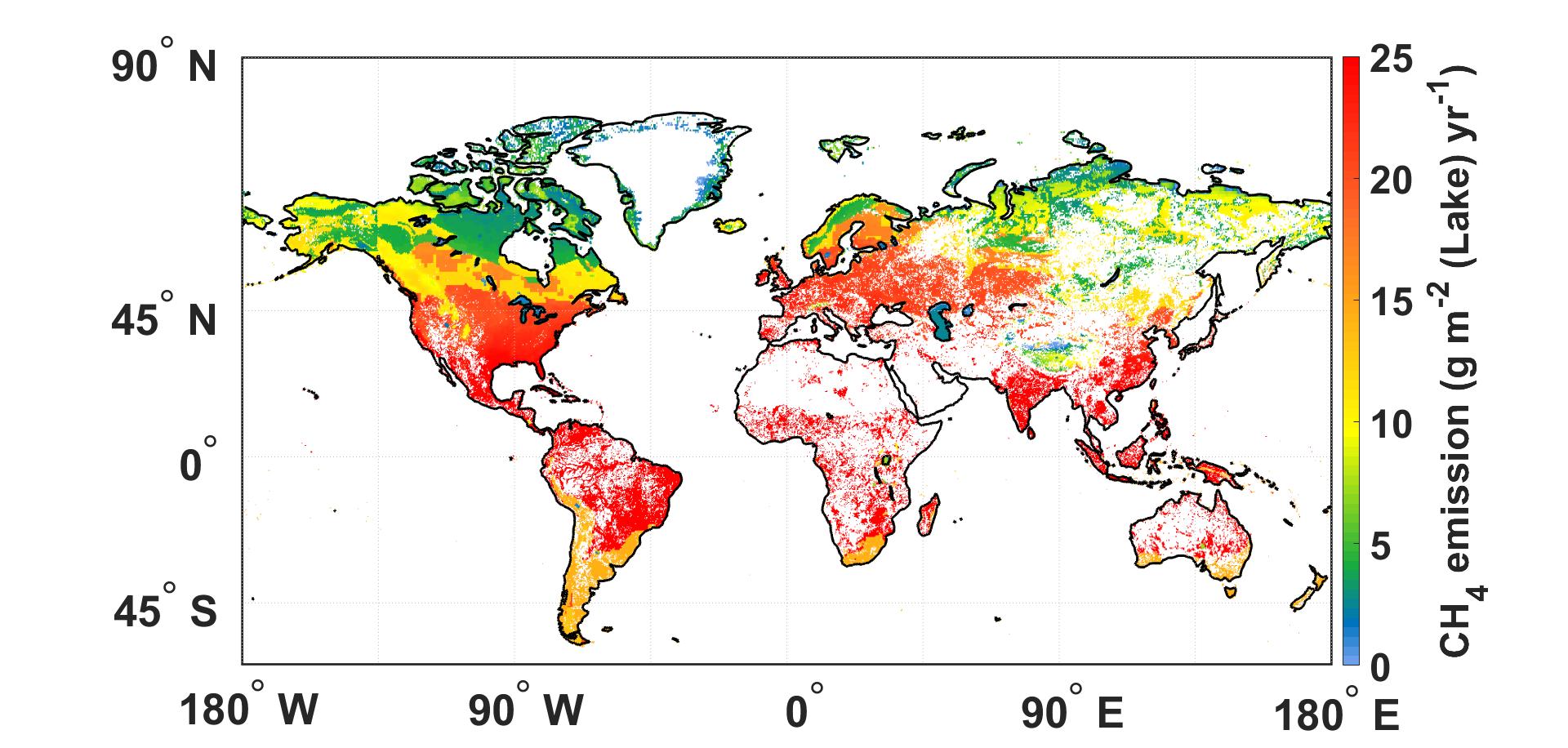


Figure S4. Global distribution of annual lake CH_4_ emission (gCH_4_ m^-2^ yr^-1^) from diffusive and ebullitive fluxes. This figure shows emissions per m^2^ of lake in each 0.25° × 0.25° grid cell.

Data Set S1. This spreadsheet is the compilation of lake CH_4_ flux measurements developed for application in this study. References for the data included in Data set S1 provided below.
